# Cannabidiol, among Other Cannabinoid Drugs, Modulates Prepulse Inhibition of Startle in the SHR Animal Model: Implications for Schizophrenia Pharmacotherapy

**DOI:** 10.3389/fphar.2016.00303

**Published:** 2016-09-09

**Authors:** Fernanda F. Peres, Raquel Levin, Valéria Almeida, Antonio W. Zuardi, Jaime E. Hallak, José A. Crippa, Vanessa C. Abilio

**Affiliations:** ^1^Interdisciplinary Laboratory of Clinical Neurosciences, Department of Psychiatry, Escola Paulista De Medicina, Federal University of São PauloSão Paulo, Brazil; ^2^Department of Pharmacology, Escola Paulista De Medicina, Federal University of São PauloSão Paulo, Brazil; ^3^Department of Neuroscience and Behavior, University of São PauloRibeirão Preto, Brazil; ^4^National Institute for Translational Medicine (INCT-TM, CNPq)Ribeirão Preto, Brazil

**Keywords:** prepulse inhibition of startle reflex, animal models, schizophrenia, cannabidiol, endocannabinoid system, SHR strain

## Abstract

Schizophrenia is a severe psychiatric disorder that involves positive, negative and cognitive symptoms. Prepulse inhibition of startle reflex (PPI) is a paradigm that assesses the sensorimotor gating functioning and is impaired in schizophrenia patients as well as in animal models of this disorder. Recent data point to the participation of the endocannabinoid system in the pathophysiology and pharmacotherapy of schizophrenia. Here, we focus on the effects of cannabinoid drugs on the PPI deficit of animal models of schizophrenia, with greater focus on the SHR (Spontaneously Hypertensive Rats) strain, and on the future prospects resulting from these findings.

**Schizophrenia** is a debilitating neuropsychiatric disorder that affects 0.7% of world's population (MacDonald and Schulz, 2009) and involves positive (i.e., delusions and hallucinations), negative (e.g., anhedonia, social withdrawal, affective flattening), and cognitive symptoms (such as impaired processing of information and deficits in working memory) (van Os and Kapur, 2009). Currently, schizophrenia's pharmacotherapy is mainly limited to the positive symptoms and associated with severe side effects and high rates of treatment resistance (Briles et al., 2012; Hasan et al., 2012; Abi-Dargham, 2014).

KEY CONCEPT 1 **Schizophrenia**

Debilitating psychiatric disorder that affects 0.7% of world's population and presents an onset between late adolescence and early adulthood. Schizophrenia's symptomatology includes positive, negative and cognitive symptoms.

**Sensorimotor gating** is a physiological process that filters sensory information as it is transmitted to motor output systems, preventing information overload and cognitive fragmentation (Cryan and Reif, 2012). This process is impaired especially in schizophrenia (Braff et al., 2001), but also in other neuropsychiatric disorders such as obsessive-compulsive disorder (Ahmari et al., 2012), Tourette's syndrome (Swerdlow et al., 2001), Huntington's disease (Swerdlow et al., 1995), and bipolar disorder (Perry et al., 2001). **Prepulse inhibition of startle reflex (PPI)** is considered an operational measure of sensorimotor gating and is extensively used in translational studies of schizophrenia, since it is seen in both rodents and humans (Braff et al., 2001). PPI is defined as a reduction of acoustic startle reflex to an intense stimulus (pulse) when immediately preceded by a low intensity stimulus (prepulse).

KEY CONCEPT 2 **Sensorimotor gating**

Physiological process that filters sensory information as it is transmitted to motor output systems, preventing information overload and cognitive fragmentation. It is impaired in schizophrenia and in other neuropsychiatric disorders.

KEY CONCEPT 3 **Prepulse inhibition of startle reflex (PPI)**

Reduction of acoustic startle reflex to an intense stimulus (pulse) when immediately preceded by a low intensity stimulus (prepulse). PPI is an operational measure of sensorimotor gating and is seen in both rodents and humans, being extensively used in translational studies.

Prepulse inhibition of startle reflex (PPI) is disrupted in schizophrenia patients and evidence show that PPI deficits are positively correlated to thought disorder (Perry and Braff, 1994; Perry et al., 1999), and associated with impaired functional status and with the presence of auditory hallucinations (Swerdlow et al., 2006; Kumari et al., 2008). PPI deficits are improved by treatment with **antipsychotic drugs** (Kumari et al., 1999; Weike et al., 2000; Leumann et al., 2002; Oranje et al., 2002; Minassian et al., 2007; Wynn et al., 2007; Martinez-Gras et al., 2009), and this improvement is associated with treatment-related amelioration of schizophrenia symptoms (Minassian et al., 2007). PPI is also disrupted in several animal models of this disorder (Swerdlow et al., 2008), being a useful paradigm to investigate the neurobiology and pharmacotherapy of information processing abnormalities in schizophrenia.

KEY CONCEPT 4 **Antipsychotic drugs**

Drugs used primarily to treat psychotic states, in particular schizophrenia and bipolar disorder. The antipsychotic drugs are classified in typical and atypical compounds: the typical antipsychotic drugs are associated with motor side effects, and the atypical are linked to metabolic disturbances.

Recent data point to the involvement of the **Endocannabinoid system** in the pathophysiology of schizophrenia. The endocannabinoid system was described subsequent to the identification of the molecular target of Δ^9^-tetrahydrocannabinol (Δ^9^-THC), the main psychoactive compound of *Cannabis sativa*, and comprises the classical cannabinoid-1 and 2 receptors (CB_1_ and CB_2_), their endogenous ligands known as endocannabinoids (e.g., anandamide and 2-arachidonoylglycerol), and the enzymes involved in the endocannabinoid's synthesis and degradation.

KEY CONCEPT 5 **Endocannabinoid system**

System described subsequent to the identification of the molecular target of Δ^9^-THC. It comprises the cannabinoid-1 and 2 receptors (CB_1_ and CB_2_), their endogenous ligands (named endocannabinoids) and the enzymes involved in the endocannabinoid's metabolism.

The contribution of the endocannabinoid system in schizophrenia is suggested based on some compelling evidence. The exposure to Δ^9^-THC may induce a transient psychotic condition in healthy subjects (D'Souza et al., 2004; Morrison et al., 2009), whereas in schizophrenia patients, cannabis consumption provokes more and earlier psychotic relapses, even among those under antipsychotic treatment (Linszen et al., 1994; D'Souza et al., 2005; Grech et al., 2005). Moreover, cannabis use has been proved to be a risk factor for psychotic outcomes (Matheson et al., 2011). In accordance, several alterations in the endocannabinoid system are seen in schizophrenia: (1) levels of anandamide are increased in patients' cerebrospinal fluid and peripheral blood (Leweke et al., 1999, 2007; De Marchi et al., 2003; Giuffrida et al., 2004); (2) *post-mortem* studies show increased CB_1_ density in patients' dorsolateral prefrontal, anterior cingulate and posterior cingulate cortices (Dean et al., 2001; Zavitsanou et al., 2004; Newell et al., 2006; Dalton et al., 2011); (3) *in vivo* studies using positron emission tomography (PET) reveal increased density of CB_1_ in the brain in both medicated and non-medicated schizophrenia patients (Wong et al., 2010; Ceccarini et al., 2013); (4) polymorphisms of the genes that code the cannabinoid receptors CB_1_ and CB_2_ are associated to some schizophrenia phenotypes (Ujike et al., 2002; Chavarría-Siles et al., 2008; Ishiguro et al., 2010). In addition, pre-clinical studies show that cannabinoid drugs are able to modulate schizophrenia-like behaviors, including PPI.

In the work that generated this focused review, we evaluated the effects of four cannabinoid drugs on the PPI deficit displayed by the **Spontaneously Hypertensive Rat (SHR) strain** an animal model characterized by our group to study several aspects of schizophrenia (Levin et al., 2014; Table 1). Here, we will focus on the effects of these four cannabinoid drugs—WIN 55212,2 (WIN–cannabinoid agonist), rimonabant (CB_1_ antagonist/inverse agonist), AM404 (anandamide uptake inhibitor) and cannabidiol (CB_1_ antagonist, anandamide uptake inhibitor, among other effects)—on PPI of animal models, mainly the SHR strain, and on the future prospects resulting from these findings.

KEY CONCEPT 6 **Spontaneously hypertensive rat (SHR) strain**

Inbred strain developed by brother-sister mating rats with hypertensive phenotype of the outbred strain Wistar. SHRs display spontaneous hypertension and also several behavioral abnormalities that fit the behavioral phenotype associated with schizophrenia.

**Table 1:** **Cannabinoid drugs as potential agents to treat prepulse inhibition of startle (PPI) deficits in schizophrenia**.

**Cannabinoid drug**	**Effect on SHR's PPI**	**Limitations/advantages**
WIN 55212,2	Attenuates the deficit	Induces psychotomimetic effects in animal models.
Rimonabant	Worsens the deficit	Is associated with increased symptoms of depression and anxiety.
AM 404	Does not modify PPI	
Cannabidiol	Attenuates the deficit	Displays antipsychotic properties in humans and in other behavioral abnormalities in animal models. Is safe in animals and humans.

SHR strain was developed by selecting rats from Wistar strain with hypertensive phenotype and brother-sister mating (Okamoto and Aoki, 1963). In addition to hypertension, the inbreeding selected some behavioral abnormalities—such as impulsivity, impaired sustained attention and hyperactivity—leading to the proposal of the SHR strain (mainly young animals) as an animal model for attention deficit/hyperactivity disorder (ADHD). It is noteworthy that most of the studies using SHR as ADHD model were performed using as control the Wistar-Kyoto strain (developed by inbreeding Wistar rats without hypertension). Wistar-Kyoto rats may be inappropriate as a control strain since they display inactivity and depressive-like behavior when compared to Wistar rats (WRs) (Overstreet, 2012), and do not show genetic similarities when compared to SHRs (Johnson et al., 1992; St Lezin et al., 1992). In addition, SHR's predictive validity as ADHD model is inconsistent: several studies describe that the administration of psychostimulants (drugs used to treat ADHD) does not attenuate SHRs' behavioral abnormalities (Amini et al., 2004; Yang et al., 2006; van den Bergh et al., 2006; Bizot et al., 2007; Barron et al., 2009; Calzavara et al., 2009), and may even potentiate them (Amini et al., 2004; Yang et al., 2006; Barron et al., 2009; Calzavara et al., 2009).

Conversely, we reported that SHRs, when compared to WRs, display schizophrenia-like behavioral abnormalities. SHRs display increased locomotion (a model for the positive symptoms of schizophrenia—Lipska and Weinberger, 2000), decreased social interaction (that mimics the negative symptoms—File and Seth, 2003), and deficits in the contextual fear conditioning (associated with impairments in emotional memory seen in schizophrenia—Maren et al., 2013) and PPI. These abnormalities are reversed by antipsychotic drugs (with varied effects in WRs, depending on the drug, the dose, and the behavior), but not by psychostimulant drugs, mood stabilizers, dopaminergic antagonists without antipsychotic activity or drugs that modulate anxiety (Calzavara et al., 2009, 2011; Levin et al., 2011). In addition, psychotomimetic manipulations, such as sleep deprivation and administration of psychostimulants, potentiate the behavioral abnormalities displayed by SHRs and induce a schizophrenia-like behavioral phenotype in WRs (Calzavara et al., 2009, 2011; Levin et al., 2011)—as seen in other animal models of the disorder, as well as in patients (Laruelle et al., 1999; Jones et al., 2011). As a result, the SHR strain has been used to investigate genetic alterations related to schizophrenia as well as novel therapeutic strategies for this disorder, including cannabinoid drugs (Levin et al., 2012, 2014; Almeida et al., 2013, 2014; Diana et al., 2015).

WIN is an agonist of CB_1_ and CB_2_ receptors. The acute administration of WIN attenuates the SHRs' PPI deficit, and does not modify the PPI of WRs—although a trend to reduce the WRs' PPI is seen with the lowest dose (Levin et al., 2014). Accordingly, other studies demonstrate that WIN reverses the PPI impairment displayed by psychosocially stressed mice (Brzózka et al., 2011) and induced by chronic administration phencyclidine (Spano et al., 2010), two other animal models of schizophrenia. The beneficial effect of WIN on the PPI of psychosocially stressed mice is prevented by pretreatment with the CB_1_ antagonist rimonabant (Brzózka et al., 2011), suggesting an involvement of these receptors on the WIN's actions on sensorimotor gating functioning.

In rodents without PPI deficits some authors describe absence of WIN effect on PPI (Bortolato et al., 2005; Brzózka et al., 2011), but others show that WIN disrupts PPI when administered systemically (Schneider and Koch, 2002; Wegener et al., 2008; Brosda et al., 2011), intra-prefrontal cortex, or intra-ventral hippocampus (Wegener et al., 2008). In addition, repeated administration of WIN during puberty induces PPI deficits that last until adulthood (Schneider and Koch, 2003; Schneider et al., 2005; Wegener and Koch, 2009; Klein et al., 2013). Therefore, WIN seems to present a psychotomimetic profile (supported also by its effects on other schizophrenia-like symptoms—Schneider and Koch, 2002; Pamplona and Takahashi, 2006; Wegener et al., 2008; Spano et al., 2010; Almeida et al., 2014), being unsuitable for schizophrenia patients.

The administration of the CB_1_ antagonist rimonabant worsens SHRs' PPI deficit, and does not alter PPI levels in WRs (Levin et al., 2014). The absence of rimonabant effects in WRs is corroborated by previous studies with animals without PPI impairments (Martin et al., 2003; Malone et al., 2004; Malone and Taylor, 2006; Ballmaier et al., 2007). In animal models of schizophrenia, while some studies show that rimonabant is able to counteract the PPI impairments (Malone et al., 2004; Nagai et al., 2006; Ballmaier et al., 2007), others show no effect (Martin et al., 2003; Malone and Taylor, 2006). In addition, clinical trials show that rimonabant induces significant psychiatric side effects, such as symptoms of depression and anxiety, and increases suicide-related adverse events (Christensen et al., 2007; Food and Drug Administration Advisory Committee, 2007; Topol et al., 2010). These data, thus, argue against the clinical use of this compound.

It should be noted that both a CB_1_/CB_2_ agonist and a CB_1_ antagonist provoke differential effects in WRs and SHRs (Levin et al., 2014). This information suggests that these rat strains display distinct endocannabinoid system functioning. Indeed, our group observed that SHRs present higher CB_1_ density in the prefrontal and anterior cingulate cortices when compared to WRs (Almeida et al., submitted), which is in accordance with data from schizophrenia patients (Dean et al., 2001; Zavitsanou et al., 2004; Newell et al., 2006; Wong et al., 2010; Dalton et al., 2011; Ceccarini et al., 2013).

AM 404 is a competitive and selective inhibitor of anandamide transportation, and therefore increases its extracellular levels. Anandamide is an endocannabinoid that acts as an agonist of CB_1_ and CB_2_ receptors and of vanilloid receptor 1 (TRPV1). When administered to WRs and SHRs, AM 404 did not modify their PPI levels (Levin et al., 2014). No other study has investigated the effects of AM 404 on PPI of animal models of schizophrenia. In control animals, one study shows absence of AM 404 effects on PPI in Sprague-Dawley rats (Bortolato et al., 2006), but another reveals that AM 404, either injected acutely or chronically, disrupts PPI in Swiss mice (Fernandez-Espejo and Galan-Rodriguez, 2004). Evidence, therefore, do not support the use of AM 404 as a strategy to treat sensorimotor processing deficits associated to schizophrenia.

It is worth mentioning that the doses of AM 404 used in our previous study have been shown to increase anandamide levels in plasma and brain regions of rats (Giuffrida et al., 2000; Bortolato et al., 2006). Clinical findings show that the levels of anandamide in the cerebrospinal fluid of non-medicated schizophrenia patients are negatively correlated to their psychotic symptoms (De Marchi et al., 2003; Giuffrida et al., 2004), and that prodromal individuals with lower levels of anandamide display a higher risk for transitting to psychosis earlier (Koethe et al., 2009). These results led some authors to suggest that anandamide plays a protective role in schizophrenia. The absence of AM 404 effects on the PPI of SHRs suggest that increasing anandamide levels is not sufficient to restore SHRs' PPI impairment.

**Cannabidiol** is one of the major constituent of cannabis, being the most abundant after Δ^9^-THC. Cannabidiol acts as an antagonist of the cannabinoid receptors CB_1_ and CB_2_ agonists and inhibits the reuptake and degradation of anandamide. It is also an antagonist of the orphan receptor GPR55 and an agonist of the serotonin receptor 5-HT_1A_ and of the vanilloid receptors TRPV1 and TRPV2 (Izzo et al., 2009). When administered to SHRs, cannabidiol restores the PPI deficits (Levin et al., 2014). In fact, this compound has been shown to restore the PPI impairments provoked by MK-801 (Long et al., 2006), and by systemic or intra-accumbens administration of amphetamine (Pedrazzi et al., 2015). In addition, when administered repeatedly, cannabidiol prevents the PPI disruption induced by chronic administration of MK-801 (Gomes et al., 2015). Acute or chronic administration of psychostimulant drugs such as MK-801 and amphetamine are used to model schizophrenia. The results, thus, point to an antipsychotic profile of cannabidiol and to the use of this compound on the treatment of sensorimotor gating impairments seen in schizophrenia.

KEY CONCEPT 7 **cannabidiol**

One of the over 60 compounds of *Cannabis sativa*, being the most abundant after Δ^9^-THC. Unlike Δ^9^-THC, cannabidiol is a non-psychotomimetic drug, and presents antipsychotic, anxiolytic, anti-inflammatory and neuroprotective actions.

In animals without PPI impairments, some authors describe that cannabidiol does not modify the PPI levels when administered acutely or chronically (Long et al., 2006; Gomes et al., 2015; Pedrazzi et al., 2015), while one study shows that it is able to disrupt PPI (Gururajan et al., 2011). Nonetheless, in our previous work, administration of cannabidiol increased PPI in WRs (Levin et al., 2014). Interestingly, typical and atypical antipsychotic drugs have been shown to increase PPI in WRs and Sprague-Dawley rats (Hoffman et al., 1993; Swerdlow and Geyer, 1993; Johansson et al., 1995; Depoortere et al., 1997a,b; Levin et al., 2011). This effect is mainly seen when PPI levels are below 50%, which is our case. Therefore, the effects of cannabidiol on PPI of WRs are in accordance with the antipsychotic profile suggested for this drug.

The mechanisms whereby cannabidiol modifies PPI are still to be elucidated. Our data suggest that the increase in PPI promoted by this drug is not only due to an increase in anandamide levels, since the anandamide uptake inhibitor AM 404 does not modify PPI (Levin et al., 2014). Likewise, it is unlikely that the cannabidiol's antagonism of CB_1_ receptors is the mechanism responsible for its effect on the PPI deficit of SHRs, given that the CB_1_ antagonist rimonabant promoted an opposite outcome (Levin et al., 2014). Regarding cannabidiol's action on CB_1_ receptors, it is noteworthy that although this compound is able to antagonize cannabinoid CB_1_/CB_2_ receptor agonists-induced stimulation in brain membranes, this effect is observed with K_B_ values in the nanomolar range, way below the Ki for displacement of [^3^H]CP55940 from CB_1_ and CB_2_ (Pertwee, 2008). In addition, the effectiveness of cannabidiol in inhibiting [^35^S]GTPγS binding to brain membranes of wild-type mouse is not different from CB1^−/−^ mouse (Pertwee, 2008). On the other hand, Long et al. (2006) described that cannabidiol's ability of restoring the PPI deficit induced by MK-801 is prevented by pretreatment with capsazepine, a TRPV1 antagonist. Thus, although other mechanisms of action cannot be disregarded, cannabidiol's effects on PPI seem to be at least partially related to its action on the vanilloid system.

Other animal studies, investigating additional schizophrenia-like behavioral paradigms, support cannabidiol's antipsychotic properties (Table 3). The administration of this compound is able to diminish the stereotyped behavior and the hyperlocomotion—behaviors that model the positive symptoms of schizophrenia—induced by psychotomimetic drugs (Zuardi et al., 1991; Moreira and Guimaraes, 2005; Long et al., 2010; Gururajan et al., 2012). Cannabidiol also restores drug-induced impairments in social interaction (Malone et al., 2009; Gururajan et al., 2012), and counteracts the deficit in contextual fear conditioning displayed by the SHRs (Levin et al., 2012).

**Table 2:** **Summary of the studies investigating the effects of cannabinoid drugs on the PPI of animal models**.

**Reference**	**Results**
**WIN 55, 212-2 (WIN)**	
Schneider and Koch, 2002	Acute administration of WIN (0.6 or 1.2 mg/kg) impairs PPI in a dose-dependent manner. The administration of haloperidol reverses the PPI deficit.
Schneider and Koch, 2003; Schneider et al., 2005	Treatment for 25 days with WIN (1.2 mg/kg) during puberty induces PPI deficits that last until adulthood. This impairment is reversed by the administration of haloperidol.
Bortolato et al., 2005	Chronic (during 7 or 21 days) or acute treatment with WIN (0.5, 1, or 2 mg/kg) does not alter PPI levels.
Wegener et al., 2008	Acute systemic administration of WIN (1.2 mg/kg), as well as the administration intra-medial prefrontal cortex or intra-dorsal hippocampus (5 μg/0.3 μl) diminish PPI levels.
Wegener and Koch, 2009	Treatment for 25 days with WIN (1.2 mg/kg) during puberty induces PPI deficits that last until adulthood. In addition, WIN treated animals display altered basal neuronal activity and respond differently to haloperidol and apomorphine.
Spano et al., 2010	Chronic WIN self-administration (12.5 μg/kg/infusion) as well as experimenter-given (0.3 mg/kg, i.v.) attenuates phencyclidine-induced impairments in PPI.
Brosda et al., 2011	Acute systemic administration of WIN (0.6 or 1.2 mg/kg) impairs PPI.
Brzózka et al., 2011	WIN (3 mg/kg) administration restores the PPI deficit induced by chronic psychosocial stress. This effect is antagonized by pretreatment with rimonabant.
Klein et al., 2013	Chronic treatment with WIN (1.2 mg/kg) during puberty induces PPI deficits that last until adulthood and are reversed by deep brain stimulation.
Levin et al., 2014	Acute administration of WIN (1 mg/kg) restores the PPI deficit displayed by the SHR strain. WIN (0.3, 1 or 3 mg/kg) does not alter the PPI of control animals.
**RIMONABANT**	
Martin et al., 2003	Acute administration of rimonabant (5 mg/kg) does not alter PPI on its own or following disruptions by apomorphine, d-amphetamine or MK-801.
Malone et al., 2004	Rimonabant (1 or 3 mg/kg) does not alter PPI on its own. The acute administration of rimonabant (3 mg/kg) inhibits the PPI disruption promoted by apomorphine.
Malone and Taylor, 2006	Acute administration of rimonabant (5 mg/kg) is not able to counteract the PPI deficit promoted by social isolation. In addition, rimonabant does not alter the PPI of control animals.
Nagai et al., 2006	Acute administration of rimonabant (10 mg/kg) reverses the Δ^9^-THC-induced PPI deficit and increased dopamine release in the nucleus accumbens.
Ballmaier et al., 2007	Acute administration of rimonabant (0.75, 1.5, or 3.0 mg/kg) does not alter PPI on its own, and counteracts the PPI disruption induced by administration of phencyclidine, MK-801 or apomorphine.
Levin et al., 2014	Acute administration of rimonabant (0.75 mg/kg) worsens the PPI deficit displayed by the SHR strain. Rimonabant (0.75, 1.5, or 3 mg/kg) does not alter the PPI of control animals.
**AM 404**	
Fernandez-Espejo and Galan-Rodriguez, 2004	AM 404 either injected acutely (2.5 mg/kg) or chronically (5 mg/kg daily, 7 days) disrupts PPI. This effect is blocked by pretreatment with rimonabant.
Bortolato et al., 2006	Acute administration of AM 404 (2.5, 5, or 10 mg/kg) does not alter PPI levels.
Levin et al., 2014	Acute administration of AM 404 (1, 5, or 10 mg/kg) does not alter the PPI of SHRs or Wistar rats.
**CANNABIDIOL**	
Long et al., 2006	Acute administration of cannabidiol (1, 5, or 15 mg/kg) does not alter PPI on its own, but reverses (5 mg/kg) the MK-801-induced disruption of PPI. Pretreatment with capsazepine (antagonist of TRPV1 receptors) prevents cannabidiol effect.
Gururajan et al., 2011	Acute administration of cannabidiol (3, 10, or 30 mg/kg) disrupts PPI on its own, and has no effect on MK-801-induced PPI disruption.
Levin et al., 2014	Acute administration of cannabidiol (30 mg/kg) restores the PPI deficit displayed by the SHR strain. Cannabidiol administration also increases the PPI levels of control animals.
Gomes et al., 2015	Treatment with MK-801 for 28 days impairs PPI. Chronic treatment with cannabidiol (30 or 60 mg/kg) attenuates this impairment. Cannabidiol does not alter PPI on its own.
Pedrazzi et al., 2015	Pretreatment with cannabidiol (15, 30, or 60 mg/kg) attenuates the amphetamine-induced disruption of PPI. Cannabidiol does not alter PPI on its own.

*MK-801, dizocilpine; PPI, prepulse inhibition of startle; SHR, spontaneously hypertensive rats; $\Delta_{-}^{9}$THC, delta-9-tetrahydrocannabinol; WIN, WIN 55212,2*.

**Table 3:** **Antipsychotic effects of cannabidiol in psychiatric patients and in schizophrenia-like behaviors in animal models (for PPI, see Table 2)**.

**Reference**	**Results**
**ANIMAL MODELS**	
Zuardi et al., 1991	Acute administration of cannabidiol (60 mg/kg) diminishes the stereotyped behavior induced by apomorphine, without promoting catalepsy.
Moreira and Guimaraes, 2005	Acute administration of cannabidiol (30 or 60 mg/kg) attenuates the hyperlocomotion induced by d-amphetamine, without promoting catalepsy.
Malone et al., 2009	Pretreatment with cannabidiol (20 mg/kg) counteracts the Δ^9^-THC-induced decrease in social interaction.
Long et al., 2010	Chronic treatment with cannabidiol (50 mg/kg, 21 days) attenuates the dexamphetamine-induced hyperlocomotion.
Gururajan et al., 2012	Pretreatment with cannabidiol (3 mg/kg) counteracts the hyperlocomotion and the decrease in social interaction induced by MK-801.
Levin et al., 2012	Acute administration of cannabidiol (1 mg/kg) restores the SHR's deficit in the contextual fear conditioning task.
**PSYCHIATRIC PATIENTS**	
Zuardi et al., 1995	Treatment with cannabidiol for 4 weeks reduced the psychotic symptoms in one schizophrenia patient.
Zuardi et al., 2009	Treatment with cannabidiol for 4 weeks, in addition to their usual treatment, reduced the psychotic symptoms in six patients with Parkinson's disease without worsening their motor function.
Leweke et al., 2012	Treatment with cannabidiol for 4 weeks reduced the schizophrenia symptoms in 21 schizophrenia patients, in a way non-inferior to the antipsychotic amisulpride. Cannabidiol induced fewer side effects than amisulpride.
GW Pharmaceuticals, 2015	Proof of concept study including 88 schizophrenia patients. Treatment with cannabidiol for 6 weeks, in addition to their usual antipsychotic medication, reduced the schizophrenia symptoms without inducing serious adverse events.

*MK-801, dizocilpine; SHR, spontaneously hypertensive rats; $\Delta_{-}^{9}$THC, delta-9-tetrahydrocannabinol*.

The promising aforementioned pre-clinical data led to clinical studies (Table 3). In healthy volunteers, cannabidiol is able to attenuate the psychotic symptoms induced by the administration of psychotomimetic drugs (Karniol et al., 1974; Zuardi et al., 1982; Bhattacharyya et al., 2010). In a case-study, this cannabinoid significantly reduced schizophrenia symptoms in the Brief Psychiatric Rating Scale (BPRS), in a way superior to the typical antipsychotic drug haloperidol (Zuardi et al., 1995). Moreover, an open-label study with Parkinson's disease patients showed that cannabidiol administration, in addition to their usual treatment, decreases psychotic symptoms—evaluated by BPRS and by the Parkinson Psychosis Questionnaire—without worsening their motor function (Zuardi et al., 2009). Also, several studies suggest that cannabidiol is safe in humans and animals, and does not alter physiological parameters (blood pressure, heart rate and body temperature) or induce motor and psychological side effects (Bergamaschi et al., 2011).

Thereafter, a double-blind controlled clinical trial with schizophrenia patients was performed: treatment with cannabidiol, for 4 weeks, decreased patients symptoms—evaluated by BPRS and Positive and Negative Syndrome Scale (PANSS)—in a way non-inferior to amisulpride, one of the most effective antipsychotic drugs currently in use. Interestingly, the cannabinoid induced fewer side effects (weight gain, changes in prolactin levels and extrapyramidal symptoms) than amisulpride, and treatment with cannabidiol, but not with amisulpride, led to an increase in the levels of anandamide in serum that was associated with the decrease of psychotic symptoms (Leweke et al., 2012).

All these encouraging clinical and pre-clinical data led to a recent proof-of-concept study by GW Pharmaceuticals. The phase IIa included 88 schizophrenia patients only partially responsive to standard antipsychotic treatment, who received cannabidiol or placebo in addition to their antipsychotic medication for 6 weeks. Cannabidiol was consistently superior to placebo in attenuating the schizophrenia symptoms, and did not induce serious adverse events (GW Pharmaceuticals, 2015).

Taken as a whole, data regarding the effects of cannabinoid drugs on PPI reinforce the involvement of the endocannabinoid system in the sensorimotor gating functioning and in the pathophysiology of schizophrenia. Among the drugs that act on the endocannabinoid system, pre-clinical and the subsequent clinical data point to cannabidiol as the most promising compound for treating schizophrenia symptoms without inducing significant side effects. Nevertheless, most of the clinical evidence that suggests cannabidiol as a new antipsychotic agent or adjunctive treatment does not investigate specifically sensorimotor gating/ PPI deficits. Accordingly, data from pre-clinical studies using animal models, like our previous one (Levin et al., 2014), are fundamental to support future clinical studies focused on these deficits in schizophrenia patients.

## Author contributions

All authors listed, have made substantial, direct and intellectual contribution to the work, and approved it for publication.

## Funding

This work was supported by grants of “Fundação de Amparo à Pesquisa do Estado de São Paulo–FAPESP” and of “Conselho Nacional do Desenvolvimento Científico e Tecnológico–CAPES.”

### Conflict of interest statement

JH, AZ, and JC are co-inventors of the patent “Fluorinated CBD compounds, compositions and uses thereof. Pub. No.: WO/2014/108899. International Application No.: PCT/IL2014/050023.” The Other authors declare that the research was conducted in the absence of any commercial or financial relationships that could be construed as a potential conflict of interest.

## References

[B1] Abi-Dargham A. (2014). Schizophrenia: overview and dopamine dysfunction. J. Clin. Psychiatry 75:e31. 10.4088/JCP.13078tx2c 25470107

[B2] Ahmari S. E. Risbrough V. B. Geyer M. A. Simpson H. B. (2012). Impaired sensorimotor gating in unmedicated adults with obsessive-compulsive disorder. Neuropsychopharmacology 37, 1216–1223. 10.1038/npp.2011.308 22218093 PMC3306882

[B3] Almeida V. Levin R. Peres F. F. Niigaki S. T. Calzavara M. B. Zuardi A. W.. (2013). Cannabidiol exhibits anxiolytic but not antipsychotic property evaluated in the social interaction test. Prog. Neuropsychopharmacol. Biol. Psychiatry 41, 30–35. 10.1016/j.pnpbp.2012.10.024 23127569

[B4] Almeida V. Peres F. F. Levin R. Suiama M. A. Calzavara M. B. Zuardi A. W.. (2014). Effects of cannabinoid and vanilloid drugs on positive and negative-like symptoms on an animal model of schizophrenia: the SHR strain. Schizophr. Res. 153, 150–159. 10.1016/j.schres.2014.01.039 24556469

[B5] Amini B. Yang P. B. Swann A. C. Dafny N. (2004). Differential locomotor responses in male rats from three strains to acute methylphenidate. Int. J. Neurosci. 114, 1063–1084. 10.1080/00207450490475526 15370174

[B6] Ballmaier M. Bortolato M. Rizzetti C. Zoli M. Gessa G. Heinz A.. (2007). Cannabinoid receptor antagonists counteract sensorimotor gating deficits in the phencyclidine model of psychosis. Neuropsychopharmacology 32, 2098–2107. 10.1038/sj.npp.1301344 17299506

[B7] Barron E. Yang P. B. Swann A. C. Dafny N. (2009). Adolescent and adult male spontaneous hyperactive rats (SHR) respond differently to acute and chronic methylphenidate (Ritalin). Int. J. Neurosci. 119, 40–58. 10.1080/00207450802330546 19116830

[B8] Bergamaschi M. M. Queiroz R. H. Zuardi A. W. Crippa J. A. (2011). Safety and side effects of cannabidiol, a *Cannabis sativa* constituent. Curr. Drug Saf. 6, 237–249. 10.2174/157488611798280924 22129319

[B9] Bhattacharyya S. Morrison P. D. Fusar-Poli P. Martin-Santos R. Borgwardt S. Winton-Brown T.. (2010). Opposite effects of delta-9-tetrahydrocannabinol and cannabidiol on human brain function and psychopathology. Neuropsychopharmacology 35, 764–774. 10.1038/npp.2009.184 19924114 PMC3055598

[B10] Bizot J. C. Chenault N. Houze B. Herpin A. David S. Pothion S.. (2007). Methylphenidate reduces impulsive behaviour in juvenile Wistar rats, but not in adult Wistar, SHR and WKY rats. Psychopharmacology (Berl.) 193, 215–223. 10.1007/s00213-007-0781-4 17406857

[B11] Bortolato M. Aru G. N. Frau R. Orrù M. Luckey G. C. Boi G.. (2005). The CB receptor agonist WIN 55,212-2 fails to elicit disruption of prepulse inhibition of the startle in Sprague-Dawley rats. Psychopharmacology (Berl.) 177, 264–271. 10.1007/s00213-004-1941-4 15290008

[B12] Bortolato M. Campolongo P. Mangieri R. A. Scattoni M. L. Frau R. Trezza V.. (2006). Anxiolytic-like properties of the anandamide transport inhibitor AM404. Neuropsychopharmacology 31, 2652–2659. 10.1038/sj.npp.1301061 16541083

[B13] Braff D. L. Geyer M. A. Swerdlow N. R. (2001). Human studies of prepulse inhibition of startle: normal subjects, patient groups, and pharmacological studies. Psychopharmacology (Berl.) 156, 234–258. 10.1007/s002130100810 11549226

[B14] Briles J. J. Rosenberg D. R. Brooks B. A. Roberts M. W. Diwadkar V. A. (2012). Review of the safety of second-generation antipsychotics: are they really “atypically” safe for youth and adults? Prim. Care Companion CNS Disord. 14:11r01298. 10.4088/PCC.11r01298 23106030 PMC3466039

[B15] Brosda J. Hayn L. Klein C. Koch M. Meyer C. Schallhorn R.. (2011). Pharmacological and parametrical investigation of prepulse inhibition of startle and prepulse elicited reactions in Wistar rats. Pharmacol. Biochem. Behav. 99, 22–28. 10.1016/j.pbb.2011.03.017 21447356

[B16] Brzózka M. M. Fischer A. Falkai P. Havemann-Reinecke U. (2011). Acute treatment with cannabinoid receptor agonist WIN55212.2 improves prepulse inhibition in psychosocially stressed mice. Behav. Brain Res. 218, 280–287. 10.1016/j.bbr.2010.11.003 21070814

[B17] Calzavara M. B. Levin R. Medrano W. A. Almeida V. Sampaio A. P. Barone L. C.. (2011). Effects of antipsychotics and amphetamine on social behaviors in spontaneously hypertensive rats. Behav. Brain Res. 225, 15–22. 10.1016/j.bbr.2011.06.026 21741413

[B18] Calzavara M. B. Medrano W. A. Levin R. Kameda S. R. Andersen M. L. Tufik S.. (2009). Neuroleptic drugs revert the contextual fear conditioning deficit presented by spontaneously hypertensive rats: a potential animal model of emotional context processing in schizophrenia? Schizophr. Bull. 35, 748–759. 10.1093/schbul/sbn006 18281713 PMC2696367

[B19] Ceccarini J. De Hert M. Van Winkel R. Peuskens J. Bormans G. Kranaster L.. (2013). Increased ventral striatal CB1 receptor binding is related to negative symptoms in drug-free patients with schizophrenia. Neuroimage 79, 304–312. 10.1016/j.neuroimage.2013.04.052 23624489

[B20] Chavarría-Siles I. Contreras-Rojas J. Hare E. Walss-Bass C. Quezada P. Dassori A.. (2008). Cannabinoid receptor 1 gene (CNR1) and susceptibility to a quantitative phenotype for hebephrenic schizophrenia. Am. J. Med. Genet. B Neuropsychiatr. Genet. 147, 279–284. 10.1002/ajmg.b.30592 18186055

[B21] Christensen R. Kristensen P. K. Bartels E. M. Bliddal H. Astrup A. (2007). Efficacy and safety of the weight-loss drug rimonabant: a meta-analysis of randomised trials. Lancet 370, 1706–1713. 10.1016/S0140-673661721-8 18022033

[B22] Cryan J. F. Reif A. (2012). Behavioral Neurogenetics. Berlin; Heidelberg: Springer.

[B23] Dalton V. S. Long L. E. Weickert C. S. Zavitsanou K. (2011). Paranoid schizophrenia is characterized by increased CB1 receptor binding in the dorsolateral prefrontal cortex. Neuropsychopharmacology 36, 1620–1630. 10.1038/npp.2011.43 21471953 PMC3138655

[B24] Dean B. Sundram S. Bradbury R. Scarr E. Copolov D. (2001). Studies on [3H]CP-55940 binding in the human central nervous system: regional specific changes in density of cannabinoid-1 receptors associated with schizophrenia and cannabis use. Neuroscience 103, 9–15. 10.1016/S0306-4522(00)00552-2 11311783

[B25] De Marchi N. De Petrocellis L. Orlando P. Daniele F. Fezza F. Di Marzo V. (2003). Endocannabinoid signalling in the blood of patients with schizophrenia. Lipids Health Dis. 2:5. 10.1186/1476-511X-2-5 12969514 PMC194767

[B26] Depoortere R. Perrault G. Sanger D. J. (1997a). Potentiation of prepulse inhibition of the startle reflex in rats: pharmacological evaluation of the procedure as a model for detecting antipsychotic activity. Psychopharmacology (Berl.) 132, 366–374. 9298514 10.1007/s002130050357

[B27] Depoortere R. Perrault G. Sanger D. J. (1997b). Some, but not all, antipsychotic drugs potentiate a low level of prepulse inhibition shown by rats of the Wistar strain. Behav. Pharmacol. 8, 364–372. 9832995 10.1097/00008877-199708000-00009

[B28] Diana M. C. Santoro M. L. Xavier G. Santos C. M. Spindola L. N. Moretti P. N.. (2015). Low expression of Gria1 and Grin1 glutamate receptors in the nucleus accumbens of Spontaneously Hypertensive Rats (SHR). Psychiatry Res. 229, 690–694. 10.1016/j.psychres.2015.08.021 26296755

[B29] D'Souza D. C. Abi-Saab W. M. Madonick S. Forselius-Bielen K. Doersch A. Braley G.. (2005). Delta-9-tetrahydrocannabinol effects in schizophrenia: implications for cognition, psychosis, and addiction. Biol. Psychiatry 57, 594–608. 10.1016/j.biopsych.2004.12.006 15780846

[B30] D'Souza D. C. Perry E. MacDougall L. Ammerman Y. Cooper T. Wu Y. T.. (2004). The psychotomimetic effects of intravenous delta-9-tetrahydrocannabinol in healthy individuals: implications for psychosis. Neuropsychopharmacology 29, 1558–1572. 10.1038/sj.npp.1300496 15173844

[B31] Fernandez-Espejo E. Galan-Rodriguez B. (2004). Sensorimotor gating in mice is disrupted after AM404, an anandamide reuptake and degradation inhibitor. Psychopharmacology (Berl.) 175, 220–224. 10.1007/s00213-004-1851-5 15088080

[B32] File S. E. Seth P. (2003). A review of 25 years of the social interaction test. Eur. J. Pharmacol. 463, 35–53. 10.1016/S0014-2999(03)01273-1 12600701

[B33] Food Drug Administration Advisory Committee U. (2007). FDA Briefing Document: Zimulti (Rimonabant) Tablets, 20 mg. Rockville, MD: FSA.

[B34] Giuffrida A. Leweke F. M. Gerth C. W. Schreiber D. Koethe D. Faulhaber J.. (2004). Cerebrospinal anandamide levels are elevated in acute schizophrenia and are inversely correlated with psychotic symptoms. Neuropsychopharmacology 29, 2108–2114. 10.1038/sj.npp.1300558 15354183

[B35] Giuffrida A. Rodriguez de Fonseca F. Nava F. Loubet-Lescoulíe P. Piomelli D. (2000). Elevated circulating levels of anandamide after administration of the transport inhibitor, AM404. Eur. J. Pharmacol. 408, 161–168. 10.1016/S0014-2999(00)00786-X 11080522

[B36] Gomes F. V. Issy A. C. Ferreira F. R. Viveros M. P. Del Bel E. A. Guimarães F. S. (2015). Cannabidiol attenuates sensorimotor gating disruption and molecular changes induced by chronic antagonism of NMDA receptors in mice. Int. J. Neuropsychopharmacol. 18, 1–10. 10.1093/ijnp/pyu041 25618402 PMC4376539

[B37] Grech A. Van Os J. Jones P. B. Lewis S. W. Murray R. M. (2005). Cannabis use and outcome of recent onset psychosis. Eur. Psychiatry 20, 349–353. 10.1016/j.eurpsy.2004.09.013 16018929

[B38] Gururajan A. Taylor D. A. Malone D. T. (2011). Effect of cannabidiol in a MK-801-rodent model of aspects of schizophrenia. Behav. Brain Res. 222, 299–308. 10.1016/j.bbr.2011.03.053 21458498

[B39] Gururajan A. Taylor D. A. Malone D. T. (2012). Cannabidiol and clozapine reverse MK-801-induced deficits in social interaction and hyperactivity in Sprague-Dawley rats. J. Psychopharmacol. 26, 1317–1332. 10.1177/0269881112441865 22495620

[B40] GW Pharmaceuticals (2015). GW Pharmaceuticals Announces Positive Proof of Concept Data in Schizophrenia. London: GWP Press Release.

[B41] Hasan A. Falkai P. Wobrock T. Lieberman J. Glenthoj B. Gattaz W. F.. (2012). World Federation of Societies of Biological Psychiatry (WFSBP) guidelines for biological treatment of schizophrenia, part 1: update 2012 on the acute treatment of schizophrenia and the management of treatment resistance. World J. Biol. Psychiatry 13, 318–378. 10.3109/15622975.2012.696143 22834451

[B42] Hoffman D. C. Donovan H. Cassella J. V. (1993). The effects of haloperidol and clozapine on the disruption of sensorimotor gating induced by the noncompetitive glutamate antagonist MK-801. Psychopharmacology (Berl.) 111, 339–344. 7870972 10.1007/BF02244950

[B43] Ishiguro H. Horiuchi Y. Ishikawa M. Koga M. Imai K. Suzuki Y.. (2010). Brain cannabinoid CB2 receptor in schizophrenia. Biol. Psychiatry 67, 974–982. 10.1016/j.biopsych.2009.09.024 19931854

[B44] Izzo A. A. Borrelli F. Capasso R. Di Marzo V. Mechoulam R. (2009). Non-psychotropic plant cannabinoids: new therapeutic opportunities from an ancient herb. Trends Pharmacol. Sci. 30, 515–527. 10.1016/j.tips.2009.07.006 19729208

[B45] Johansson C. Jackson D. M. Zhang J. Svensson L. (1995). Prepulse inhibition of acoustic startle, a measure of sensorimotor gating: effects of antipsychotics and other agents in rats. Pharmacol. Biochem. Behav. 52, 649–654. 8587900 10.1016/0091-3057(95)00160-x

[B46] Johnson M. L. Ely D. L. Turner M. E. (1992). Genetic divergence between the Wistar-Kyoto rat and the spontaneously hypertensive rat. Hypertension 19, 425–427. 1349001 10.1161/01.hyp.19.5.425

[B47] Jones C. A. Watson D. J. Fone K. C. (2011). Animal models of schizophrenia. Br. J. Pharmacol. 164, 1162–1194. 10.1111/j.1476-5381.2011.01386.x 21449915 PMC3229756

[B48] Karniol I. G. Shirakawa I. Kasinski N. Pfeferman A. Carlini E. A. (1974). Cannabidiol interferes with the effects of delta 9 - tetrahydrocannabinol in man. Eur. J. Pharmacol. 28, 172–177. 4609777 10.1016/0014-2999(74)90129-0

[B49] Klein J. Hadar R. Gotz T. Manner A. Eberhardt C. Baldassarri J.. (2013). Mapping brain regions in which deep brain stimulation affects schizophrenia-like behavior in two rat models of schizophrenia. Brain Stimul. 6, 490–499. 10.1016/j.brs.2012.09.004 23085443

[B50] Koethe D. Giuffrida A. Schreiber D. Hellmich M. Schultze-Lutter F. Ruhrmann S.. (2009). Anandamide elevation in cerebrospinal fluid in initial prodromal states of psychosis. Br. J. Psychiatry 194, 371–372. 10.1192/bjp.bp.108.053843 19336792

[B51] Kumari V. Peters E. R. Fannon D. Premkumar P. Aasen I. Cooke M. A.. (2008). Uncontrollable voices and their relationship to gating deficits in schizophrenia. Schizophr. Res. 101, 185–194. 10.1016/j.schres.2007.12.481 18262774 PMC2845800

[B52] Kumari V. Soni W. Sharma T. (1999). Normalization of information processing deficits in schizophrenia with clozapine. Am. J. Psychiatry 156, 1046–1051. 10.1176/ajp.156.7.1046 10401450

[B53] Laruelle M. Abi-Dargham A. Gil R. Kegeles L. Innis R. (1999). Increased dopamine transmission in schizophrenia: relationship to illness phases. Biol. Psychiatry 46, 56–72. 10394474 10.1016/s0006-3223(99)00067-0

[B54] Leumann L. Feldon J. Vollenweider F. X. Ludewig K. (2002). Effects of typical and atypical antipsychotics on prepulse inhibition and latent inhibition in chronic schizophrenia. Biol. Psychiatry 52, 729–739. 10.1016/S0006-3223(02)01344-6 12372664

[B55] Levin R. Almeida V. Peres F. F. Calzavara M. B. da Silva N. D. Suiama M. A.. (2012). Antipsychotic profile of cannabidiol and rimonabant in an animal model of emotional context processing in schizophrenia. Curr. Pharm. Des. 18, 4960–4965. 10.2174/138161212802884735 22716146

[B56] Levin R. Calzavara M. B. Santos C. M. Medrano W. A. Niigaki S. T. Abílio V. C. (2011). Spontaneously Hypertensive Rats (SHR) present deficits in prepulse inhibition of startle specifically reverted by clozapine. Prog. Neuropsychopharmacol. Biol. Psychiatry 35, 1748–1752. 10.1016/j.pnpbp.2011.06.003 21693159

[B57] Levin R. Peres F. F. Almeida V. Calzavara M. B. Zuardi A. W. Hallak J. E.. (2014). Effects of cannabinoid drugs on the deficit of prepulse inhibition of startle in an animal model of schizophrenia: the SHR strain. Front. Pharmacol. 5:10. 10.3389/fphar.2014.00010 24567721 PMC3915876

[B58] Leweke F. M. Giuffrida A. Koethe D. Schreiber D. Nolden B. M. Kranaster L.. (2007). Anandamide levels in cerebrospinal fluid of first-episode schizophrenic patients: impact of cannabis use. Schizophr. Res. 94, 29–36. 10.1016/j.schres.2007.04.025 17566707

[B59] Leweke F. M. Giuffrida A. Wurster U. Emrich H. M. Piomelli D. (1999). Elevated endogenous cannabinoids in schizophrenia. Neuroreport 10, 1665–1669. 10501554 10.1097/00001756-199906030-00008

[B60] Leweke F. M. Piomelli D. Pahlisch F. Muhl D. Gerth C. W. Hoyer C.. (2012). Cannabidiol enhances anandamide signaling and alleviates psychotic symptoms of schizophrenia. Transl. Psychiatry 2:e94. 10.1038/tp.2012.15 22832859 PMC3316151

[B61] Linszen D. H. Dingemans P. M. Lenior M. E. (1994). Cannabis abuse and the course of recent-onset schizophrenic disorders. Arch. Gen. Psychiatry 51, 273–279. 8161287 10.1001/archpsyc.1994.03950040017002

[B62] Lipska B. K. Weinberger D. R. (2000). To model a psychiatric disorder in animals: schizophrenia as a reality test. Neuropsychopharmacology 23, 223–239. 10.1016/S0893-133X00137-8 10942847

[B63] Long L. E. Chesworth R. Huang X. F. McGregor I. S. Arnold J. C. Karl T. (2010). A behavioural comparison of acute and chronic Delta9-tetrahydrocannabinol and cannabidiol in C57BL/6JArc mice. Int. J. Neuropsychopharmacol. 13, 861–876. 10.1017/S1461145709990605 19785914

[B64] Long L. E. Malone D. T. Taylor D. A. (2006). Cannabidiol reverses MK-801-induced disruption of prepulse inhibition in mice. Neuropsychopharmacology 31, 795–803. 10.1038/sj.npp.1300838 16052245

[B65] MacDonald A. W. Schulz S. C. (2009). What we know: findings that every theory of schizophrenia should explain. Schizophr. Bull. 35, 493–508. 10.1093/schbul/sbp017 19329559 PMC2669587

[B66] Malone D. T. Jongejan D. Taylor D. A. (2009). Cannabidiol reverses the reduction in social interaction produced by low dose Delta-tetrahydrocannabinol in rats. Pharmacol. Biochem. Behav. 93, 91–96. 10.1016/j.pbb.2009.04.010 19393686

[B67] Malone D. T. Long L. E. Taylor D. A. (2004). The effect of SR 141716 and apomorphine on sensorimotor gating in Swiss mice. Pharmacol. Biochem. Behav. 77, 839–845. 10.1016/j.pbb.2004.02.010 15099930

[B68] Malone D. T. Taylor D. A. (2006). The effect of Delta9-tetrahydrocannabinol on sensorimotor gating in socially isolated rats. Behav. Brain Res. 166, 101–109. 10.1016/j.bbr.2005.07.009 16143410

[B69] Maren S. Phan K. L. Liberzon I. (2013). The contextual brain: implications for fear conditioning, extinction and psychopathology. Nat. Rev. Neurosci. 14, 417–428. 10.1038/nrn3492 23635870 PMC5072129

[B70] Martin R. S. Secchi R. L. Sung E. Lemaire M. Bonhaus D. W. Hedley L. R.. (2003). Effects of cannabinoid receptor ligands on psychosis-relevant behavior models in the rat. Psychopharmacology (Berl.) 165, 128–135. 10.1007/s00213-002-1240-x 12404071

[B71] Martinez-Gras I. Rubio G. del Manzano B. A. Rodriguez-Jimenez R. Garcia-Sanchez F. Bagney A.. (2009). The relationship between prepulse inhibition and general psychopathology in patients with schizophrenia treated with long-acting risperidone. Schizophr. Res. 115, 215–221. 10.1016/j.schres.2009.09.035 19846280

[B72] Matheson S. L. Shepherd A. M. Laurens K. R. Carr V. J. (2011). A systematic meta-review grading the evidence for non-genetic risk factors and putative antecedents of schizophrenia. Schizophr. Res. 133, 133–142. 10.1016/j.schres.2011.09.020 21999904

[B73] Minassian A. Feifel D. Perry W. (2007). The relationship between sensorimotor gating and clinical improvement in acutely ill schizophrenia patients. Schizophr. Res. 89, 225–231. 10.1016/j.schres.2006.08.006 17005374 PMC2676911

[B74] Moreira F. A. Guimarães F. S. (2005). Cannabidiol inhibits the hyperlocomotion induced by psychotomimetic drugs in mice. Eur. J. Pharmacol. 512, 199–205. 10.1016/j.ejphar.2005.02.040 15840405

[B75] Morrison P. D. Zois V. McKeown D. A. Lee T. D. Holt D. W. Powell J. F.. (2009). The acute effects of synthetic intravenous Delta9-tetrahydrocannabinol on psychosis, mood and cognitive functioning. Psychol. Med. 39, 1607–1616. 10.1017/S0033291709005522 19335936

[B76] Nagai H. Egashira N. Sano K. Ogata A. Mizuki A. Mishima K.. (2006). Antipsychotics improve Delta9-tetrahydrocannabinol-induced impairment of the prepulse inhibition of the startle reflex in mice. Pharmacol. Biochem. Behav. 84, 330–336. 10.1016/j.pbb.2006.05.018 16820196

[B77] Newell K. A. Deng C. Huang X. F. (2006). Increased cannabinoid receptor density in the posterior cingulate cortex in schizophrenia. Exp. Brain Res. 172, 556–560. 10.1007/s00221-006-0503-x 16710682

[B78] Okamoto K. Aoki K. (1963). Development of a strain of spontaneously hypertensive rats. Jpn. Circ. J. 27, 282–293. 13939773 10.1253/jcj.27.282

[B79] Oranje B. Van Oel C. J. Gispen-De Wied C. C. Verbaten M. N. Kahn R. S. (2002). Effects of typical and atypical antipsychotics on the prepulse inhibition of the startle reflex in patients with schizophrenia. J. Clin. Psychopharmacol. 22, 359–365. 10.1097/00004714-200208000-00005 12172334

[B80] Overstreet D. H. (2012). Modeling depression in animal models. Methods Mol. Biol. 829, 125–144. 10.1007/978-1-61779-458-2_7 22231810

[B81] Pamplona F. A. Takahashi R. N. (2006). WIN 55212-2 impairs contextual fear conditioning through the activation of CB1 cannabinoid receptors. Neurosci. Lett. 397, 88–92. 10.1016/j.neulet.2005.12.026 16406322

[B82] Pedrazzi J. F. Issy A. C. Gomes F. V. Guimarães F. S. Del-Bel E. A. (2015). Cannabidiol effects in the prepulse inhibition disruption induced by amphetamine. Psychopharmacology (Berl.) 232, 3057–3065. 10.1007/s00213-015-3945-7 25943166

[B83] Perry W. Braff D. L. (1994). Information-processing deficits and thought disorder in schizophrenia. Am. J. Psychiatry 151, 363–367. 10.1176/ajp.151.3.363 8109644

[B84] Perry W. Geyer M. A. Braff D. L. (1999). Sensorimotor gating and thought disturbance measured in close temporal proximity in schizophrenic patients. Arch. Gen. Psychiatry 56, 277–281. 10078506 10.1001/archpsyc.56.3.277

[B85] Perry W. Minassian A. Feifel D. Braff D. L. (2001). Sensorimotor gating deficits in bipolar disorder patients with acute psychotic mania. Biol. Psychiatry 50, 418–424. 10.1016/S0006-3223(01)01184-2 11566158

[B86] Pertwee R. G. (2008). The diverse CB1 and CB2 receptor pharmacology of three plant cannabinoids: delta9-tetrahydrocannabinol, cannabidiol and delta9-tetrahydrocannabivarin. Br. J. Pharmacol. 153, 199–215. 10.1038/sj.bjp.0707442 17828291 PMC2219532

[B87] Schneider M. Drews E. Koch M. (2005). Behavioral effects in adult rats of chronic prepubertal treatment with the cannabinoid receptor agonist WIN 55,212-2. Behav. Pharmacol. 16, 447–454. 10.1097/00008877-200509000-00018 16148450

[B88] Schneider M. Koch M. (2002). The cannabinoid agonist WIN 55,212-2 reduces sensorimotor gating and recognition memory in rats. Behav. Pharmacol. 13, 29–37. 10.1097/00008877-200202000-00003 11990717

[B89] Schneider M. Koch M. (2003). Chronic pubertal, but not adult chronic cannabinoid treatment impairs sensorimotor gating, recognition memory, and the performance in a progressive ratio task in adult rats. Neuropsychopharmacology 28, 1760–1769. 10.1038/sj.npp.1300225 12888772

[B90] Spano M. S. Fadda P. Frau R. Fattore L. Fratta W. (2010). Cannabinoid self-administration attenuates PCP-induced schizophrenia-like symptoms in adult rats. Eur. Neuropsychopharmacol. 20, 25–36. 10.1016/j.euroneuro.2009.09.004 19854030

[B91] St Lezin E. Simonet L. Pravenec M. Kurtz T. W. (1992). Hypertensive strains and normotensive ‘control’ strains. How closely are they related? Hypertension 19, 419–424. 1568758 10.1161/01.hyp.19.5.419

[B92] Swerdlow N. R. Geyer M. A. (1993). Clozapine and haloperidol in an animal model of sensorimotor gating deficits in schizophrenia. Pharmacol. Biochem. Behav. 44, 741–744. 8451276 10.1016/0091-3057(93)90193-w

[B93] Swerdlow N. R. Karban B. Ploum Y. Sharp R. Geyer M. A. Eastvold A. (2001). Tactile prepuff inhibition of startle in children with Tourette's syndrome: in search of an “fMRI-friendly” startle paradigm. Biol. Psychiatry 50, 578–585. 10.1016/S0006-3223(01)01164-7 11690592

[B94] Swerdlow N. R. Light G. A. Cadenhead K. S. Sprock J. Hsieh M. H. Braff D. L. (2006). Startle gating deficits in a large cohort of patients with schizophrenia: relationship to medications, symptoms, neurocognition, and level of function. Arch. Gen. Psychiatry 63, 1325–1335. 10.1001/archpsyc.63.12.1325 17146007

[B95] Swerdlow N. R. Paulsen J. Braff D. L. Butters N. Geyer M. A. Swenson M. R. (1995). Impaired prepulse inhibition of acoustic and tactile startle response in patients with Huntington's disease. J. Neurol. Neurosurg. Psychiatr. 58, 192–200. 7876851 10.1136/jnnp.58.2.192 PMC1073317

[B96] Swerdlow N. R. Weber M. Qu Y. Light G. A. Braff D. L. (2008). Realistic expectations of prepulse inhibition in translational models for schizophrenia research. Psychopharmacology (Berl.) 199, 331–388. 10.1007/s00213-008-1072-4 18568339 PMC2771731

[B97] Topol E. J. Bousser M. G. Fox K. A. Creager M. A. Despres J. P. Easton J. D.. (2010). Rimonabant for prevention of cardiovascular events (CRESCENDO): a randomised, multicentre, placebo-controlled trial. Lancet 376, 517–523. 10.1016/S0140-673660935-X 20709233

[B98] Ujike H. Takaki M. Nakata K. Tanaka Y. Takeda T. Kodama M.. (2002). CNR1, central cannabinoid receptor gene, associated with susceptibility to hebephrenic schizophrenia. Mol. Psychiatry 7, 515–518. 10.1038/sj.mp.4001029 12082570

[B99] van den Bergh F. S. Bloemarts E. Chan J. S. Groenink L. Olivier B. Oosting R. S. (2006). Spontaneously hypertensive rats do not predict symptoms of attention-deficit hyperactivity disorder. Pharmacol. Biochem. Behav. 83, 380–390. 10.1016/j.pbb.2006.02.018 16580713

[B100] van Os J. Kapur S. (2009). Schizophrenia. Lancet 374, 635–645. 10.1016/S0140-673660995-8 19700006

[B101] Wegener N. Koch M. (2009). Behavioural disturbances and altered Fos protein expression in adult rats after chronic pubertal cannabinoid treatment. Brain Res. 1253, 81–91. 10.1016/j.brainres.2008.11.081 19094973

[B102] Wegener N. Kuhnert S. Thüns A. Roese R. Koch M. (2008). Effects of acute systemic and intra-cerebral stimulation of cannabinoid receptors on sensorimotor gating, locomotion and spatial memory in rats. Psychopharmacology (Berl.) 198, 375–385. 10.1007/s00213-008-1148-1 18446326

[B103] Weike A. I. Bauer U. Hamm A. O. (2000). Effective neuroleptic medication removes prepulse inhibition deficits in schizophrenia patients. Biol. Psychiatry 47, 61–70. 10.1016/S0006-3223(99)00229-2 10650450

[B104] Wong D. F. Kuwabara H. Horti A. G. Raymont V. Brasic J. Guevara M.. (2010). Quantification of cerebral cannabinoid receptors subtype 1 (CB1) in healthy subjects and schizophrenia by the novel PET radioligand [11C]OMAR. Neuroimage 52, 1505–1513. 10.1016/j.neuroimage.2010.04.034 20406692 PMC6580862

[B105] Wynn J. K. Green M. F. Sprock J. Light G. A. Widmark C. Reist C.. (2007). Effects of olanzapine, risperidone and haloperidol on prepulse inhibition in schizophrenia patients: a double-blind, randomized controlled trial. Schizophr. Res. 95, 134–142. 10.1016/j.schres.2007.05.039 17662577 PMC2716219

[B106] Yang P. B. Swann A. C. Dafny N. (2006). Acute and chronic methylphenidate dose-response assessment on three adolescent male rat strains. Brain Res. Bull. 71, 301–310. 10.1016/j.brainresbull.2006.09.019 17113960 PMC2048685

[B107] Zavitsanou K. Garrick T. Huang X. F. (2004). Selective antagonist [3H]SR141716A binding to cannabinoid CB1 receptors is increased in the anterior cingulate cortex in schizophrenia. Prog. Neuropsychopharmacol. Biol. Psychiatry 28, 355–360. 10.1016/j.pnpbp.2003.11.005 14751433

[B108] Zuardi A. W. Crippa J. A. Hallak J. E. Pinto J. P. Chagas M. H. Rodrigues G. G.. (2009). Cannabidiol for the treatment of psychosis in Parkinson's disease. J. Psychopharmacol. 23, 979–983. 10.1177/0269881108096519 18801821

[B109] Zuardi A. W. Morais S. L. Guimarães F. S. Mechoulam R. (1995). Antipsychotic effect of cannabidiol. J. Clin. Psychiatry 56, 485–486. 7559378

[B110] Zuardi A. W. Rodrigues J. A. Cunha J. M. (1991). Effects of cannabidiol in animal models predictive of antipsychotic activity. Psychopharmacology (Berl.) 104, 260–264. 1678894 10.1007/BF02244189

[B111] Zuardi A. W. Shirakawa I. Finkelfarb E. Karniol I. G. (1982). Action of cannabidiol on the anxiety and other effects produced by delta 9-THC in normal subjects. Psychopharmacology (Berl.) 76, 245–250. 6285406 10.1007/BF00432554

